# Text Messaging Data Collection for Monitoring an Infant Feeding Intervention Program in Rural China: Feasibility Study

**DOI:** 10.2196/jmir.2906

**Published:** 2013-12-04

**Authors:** Ye Li, Wei Wang, Michelle Helena van Velthoven, Li Chen, Josip Car, Igor Rudan, Yanfeng Zhang, Qiong Wu, Xiaozhen Du, Robert W Scherpbier

**Affiliations:** ^1^Department of Integrated Early Childhood DevelopmentCapital Institute of PediatricsBeijingChina; ^2^Global eHealth UnitDepartment of Primary Care and Public HealthImperial College LondonLondonUnited Kingdom; ^3^Centre for Population Health Sciences and Global Health AcademyUniversity of Edinburgh Medical SchoolEdinburghUnited Kingdom; ^4^Section of Health and NutritionWater, Environment and SanitationUNICEF ChinaBeijingChina

**Keywords:** text messaging, data collection, program evaluation, child nutrition sciences

## Abstract

**Background:**

An effective data collection method is crucial for high quality monitoring of health interventions. The traditional face-to-face data collection method is labor intensive, expensive, and time consuming. With the rapid increase of mobile phone subscribers, text messaging has the potential to be used for evaluation of population health interventions in rural China.

**Objective:**

The objective of this study was to explore the feasibility of using text messaging as a data collection tool to monitor an infant feeding intervention program.

**Methods:**

Participants were caregivers of children aged 0 to 23 months in rural China who participated in an infant feeding health education program. We used the test-retest method. First, we collected data with a text messaging survey and then with a face-to-face survey for 2 periods of 3 days. We compared the response rate, data agreement, costs, and participants’ acceptability of the two methods. Also, we interviewed participants to explore their reasons for not responding to the text messages and the reasons for disagreement in the two methods. In addition, we evaluated the most appropriate time during the day for sending text messages.

**Results:**

We included 258 participants; 99 (38.4%) participated in the text messaging survey and 177 (68.6%) in the face-to-face survey. Compared with the face-to-face survey, the text messaging survey had much lower response rates to at least one question (38.4% vs 68.6%) and to all 7 questions (27.9% vs 67.4%) with moderate data agreement (most kappa values between .5 and .75, the intraclass correlation coefficients between .53 to .72). Participants who took part in both surveys gave the same acceptability rating for both methods (median 4.0 for both on a 5-point scale, 1=disliked very much and 5=liked very much). The costs per questionnaire for the text messaging method were much lower than the costs for the face-to-face method: ¥19.7 (US $3.13) versus ¥33.9 (US $5.39) for all questionnaires, and ¥27.1 (US $4.31) versus ¥34.4 (US $5.47) for completed questionnaires. The main reasons for not replying were that participants did not receive text messages, they were too busy to reply, or they did not see text messages in time. The main reasons for disagreement in responses were that participants forgot their answers in the text messaging survey and that they changed their minds. We found that participants were more likely to reply to text messages immediately during 2 time periods: 8 AM to 3 PM and 8 PM to 9 PM.

**Conclusions:**

The text messaging method had reasonable data agreement and low cost, but a low response rate. Further research is needed to evaluate effectiveness of measures that can increase the response rate, especially in collecting longitudinal data by text messaging.

## Introduction

Globally, the proportions of stunting, underweight, and wasting of children younger than 5 years are estimated to be 26%, 16%, and 8%, respectively, and they have attributed to more than 40% of child deaths [1]. In China, the prevalences of underweight and stunting in children younger than 5 years are also high, amounting to 12.6% and 9.4%, respectively, in 2012 [2]. Inadequate breastfeeding and complementary feeding causes undernutrition in young children; thereby, affecting children’s survival [3]. The World Health Organization (WHO) recommends exclusive breastfeeding for 6 months and then providing safe and appropriate complementary foods with continued breastfeeding for children up to 2 years of age and beyond [4]. During the past decade, China has adopted the WHO’s feeding recommendations and implemented programs to improve feeding practices [5]. However, infant and young child feeding is still suboptimal: the proportion of infants younger than 6 months who were exclusively breastfed was only 27.6%, the proportion of infants aged 6-9 months who received complementary feeding was 43.3%, and the proportion of children aged 12-15 months who received continued breastfeeding was 37.0% [6].

Planning and management are essential for health programs to achieve high coverage of key interventions. Program monitoring aims to evaluate the extent to which activities are completed, such as training, supervision, home visits, and distribution of medicines, supplies, and counseling materials. Indicators related to availability, access, demand, and quality can be obtained through program records and routine reports. Monitoring is crucial for process evaluation of intervention programs, but often difficult to perform in rural areas because there are no adequate primary care records in paper or electronic format that could enable us to monitor indicators such as knowledge of caregivers or proportion of children with anemia who received iron supplements [7].

The quality of data collection is affected by different sources of bias, which together are referred to as the *total survey error* [8]. Total survey error consists of sampling errors and nonsampling errors. The choice of data collection mode influences the extent to which the data are affected by each type of nonsampling error (coverage error, nonresponse error, and measurement error) [9]. There are a wide range of data collection methods for both interviewer-administered questionnaires (including face-to-face and telephone interviews) and self-administered questionnaires (including mailed surveys and computer-assisted surveys) [9]. The face-to-face mode has long been recognized as the gold standard for its effectiveness in securing high levels of participation and, hence, to reduce nonresponse error [9]. However, in face-to-face surveys, respondents are more likely to modify the true answer to certain types of survey questions to present themselves in a more favorable light than in self-administered surveys [10,11]. In addition, the face-to-face mode is labor intensive, expensive, and time consuming [12], and the increased costs makes it very challenging and extremely costly to be used in large surveys [13], especially in resource-limited settings.

The number of mobile phone holders has increased rapidly worldwide, including in China. By May 2013, there were almost as many mobile phone subscriptions as people in the world (estimated 6.8 billion mobile subscriptions) [14]. In China, there were more than 1.1 billion mobile phone subscriptions in May 2013 [14]. There is a growing interest in using text messages in medical research and this could be an innovative way to collect data for self-administered interviews [15]. Previous studies have evaluated using text messaging for data collection. Haberer et al [16] showed in their qualitative study that participants expressed a high level of acceptance of text message data collection about antiretroviral therapy adherence in a resource-limited setting. Whitford et al [17] found that participants perceived text messaging as an acceptable way of providing data and that researchers found it an easy and functional method of gathering data. Other studies demonstrated that it was feasible to use text messaging to document bleeding episodes in children with hemophilia [18], to collect data on pain after tonsillectomy [19], and to collect frequent data for monitoring the clinical course of low back pain [20]. Studies also indicated that text messaging data collection has many advantages: it is accessible for many people regardless of time, place, or setting, and it can collect information in real time without interviewer bias [12]. In certain cases, it makes it possible to collect longitudinal data [21-23] and can give access to a migrating population and to other participants that are difficult to reach [24,25].

The choice between modes of data collection is guided by data quality, but also by the available organizational infrastructure, estimated costs, predicted nonresponse rate, and length of data collection period [26]. As far as we know, data collection by text messaging has not yet been studied in China. Our study describes a text messaging survey and compares text messaging to face-to-face data collection to monitor an infant feeding program in rural China. We aimed to explore the feasibility of data collection through text messaging to monitor child health programs. If this method is feasible, it can be used to monitor health programs more effectively and guide planning of successful health interventions that can improve child health.

## Methods

### Overview

This study is part of the evaluation of a large-scale child health intervention program in Zhao County, China. As part of nutrition counseling in this program, we developed a feeding calendar and distributed it to all caregivers to explain the WHO feeding recommendations.

### Study Design

All caregivers who received the calendars with information on infant feeding were eligible for recruitment if they met our inclusion criteria. We used the test-retest method [27] and asked participants to complete a survey questionnaire for program monitoring twice: first by text messaging and then by face-to-face interviews. We compared differences between the 2 data collection methods in response rate, data agreement, costs, and acceptability to caregivers. We also explored caregivers’ reasons for not replying to text messages, reasons for answer disagreement between the two methods, and the most appropriate time during the day for sending text messages.

### Study Setting

This study was conducted in all 16 village clinics in Wangxizhang Township, Zhao County, Hebei Province, China. In Zhao County, approximately 75% of the population has mobile phones and nearly all households have at least 1 mobile phone [28]. Detailed information on the study setting can be found elsewhere [29].

### Participants

Before the study, we distributed the infant feeding calendars to pregnant women and caregivers of all children aged 0-23 months in Wangxizhang Township in January 2012. We included participants according to the following criteria: (1) had a child younger than 2 years, (2) received the infant feeding calendar prior to the study, (3) provided their mobile phone number, (4) were willing to receive text messages, and (5) had a mobile phone number that was validated by our text messages from village doctors. We excluded caregivers if their mobile phone number was wrong, if they refused to participate, or if they participated in the pilot study.

### Recruitment

We asked village doctors to distribute the infant feeding calendars in their catchment areas supervised by a doctor from Zhao County Maternal and Child Health Hospital. Village doctors were established to introduce health care in rural areas in the 1960s when a village-level cooperative medical scheme was started. However, the quality of care varied widely because funding was variable and most village doctors only received a short period of training or no training at all. Nowadays, village doctors usually have at least primary school or junior high school education and they have a good relationship with villagers [30,31]. We also asked village doctors and the hospital doctor to collect demographic information of children and their caregivers, mobile phone numbers of caregivers, and their willingness to receive text messages. We obtained a list with names of children after distribution of infant feeding calendars. Then, we sent text messages to all caregivers to validate mobile phone numbers. For those caregivers who did not respond to our text messages, we asked the village doctors to visit caregivers in their homes to verify their mobile phone numbers. We paid the village doctors and the hospital doctor for their efforts. Each village doctor was paid ¥50.0 (US $7.95) for completion of their work and the hospital doctor was paid ¥50.0 (US $7.95) per day for 2 days.

### Pilot Study

Before the formal study, we conducted a pilot study on caregivers’ willingness to receive and reply to text messages. We selected a convenience sample of 38 caregivers aged 22 to 37 years, who were raising a child aged 0-23 months in our study area. After obtaining informed consent and demographic information, we sent text messages to caregivers to test the survey. We asked caregivers about their mobile phone use, experience with text messaging surveys, and how they interpreted our questions. We found that almost every caregiver (94.7%) had their own mobile phone and that almost all (94.7%) were willing to reply to the messages for research. We revised our survey’s text messages according to the caregivers’ feedback. We added more information to the first and the second message to make it more accurate, included information about the message sender, fees for replying to messages, how to reply to a text message survey, and reduced the total number of questions from 10 to 8. In addition, we made some small changes to the wording of the text messages.

### Training of Interviewers

We recruited 3 recent graduates with Bachelor’s degrees from medical universities (2010-2011) as interviewers for this study and trained them for half a day on the procedures of the face-to-face survey. We did role-play exercises with the interviewers and discussed problems they encountered to ensure they felt comfortable and confident in conducting interviews and would conduct them with consistency.

### Data Collection and Entry Process

We first asked caregivers to participate in the text messaging survey for a period of 3 days (April 15-17, 2012), and then in the face-to-face survey for a second period of 3 days (April 18-20, 2012). According to Hermann Ebbinghaus’s test, only approximately 27.8% of newly learned meaningless syllables will be remembered after 48 hours [32]. Our study was about whether caregivers received and read the feeding calendar, their perception of the feeding calendar, and their knowledge on infant feeding, which were meaningful; therefore, we prolonged the time interval to 3 days. Participants were not allowed to look up their answers during the interview. During the study, a team member recorded all the costs data and another team member checked the data.

### Text Message Method

Before the formal text messages were sent, we asked village doctors to inform participants. We first conducted the text messaging survey for 3 days. A team member used a smartphone (ME525, Motorola, Tianjin, China) with an Android 2.2 system to send text messages to all participants individually. During the first day, we sent the first question to all participants from 10 am to 8 pm. We sent the next text messages immediately after participants responded between the hours of 8 am and 12 pm. A supervisor (a team member) checked the messages individually to ensure that the messages sent out were correct and that each message had been successfully sent. We sent the messages again if they failed to be sent, if we did not receive a reply within 1 day, or if we received an unclear response. The smartphone automatically saved all original messages and the sending and receiving time. When replying to our text messages, participants were charged ¥0.1 (US $0.02) per message; for all 8 messages that needed a reply, they were charged ¥0.8 (US $0.13) at most. In the first survey message, instead of reimbursing text messages’ fees, we told participants that caregivers who completed the text message survey (responded to all 8 questions) would receive an infant recipe as a gift for their efforts after the face-to-face interview 3 days later. This was only a small incentive for caregivers; it was worth ¥2.0 (US $0.32) and the per capita annual net income of rural households was ¥7119.7 (US $1132.29) in 2012 according to the China Statistical Yearbook [33].

### Face-to-Face Method

After the text messaging survey, we did a face-to-face survey during a second period of 3 days. Village doctors informed all 258 participants (including 159 caregivers who did not participate in the text messaging survey) to gather at the village clinics for the interviews and told them the interviews were about infant feeding knowledge. Each interview was conducted in a quiet place where an interviewer asked each participant questions and recorded answers using pen and paper. Each interview lasted approximately 8 to 10 minutes. After the face-to-face survey, a team member checked if the participant had responded to the text messaging survey and if the results were the same for both surveys. Then, interviewers asked the participants questions about their reasons for not replying and about any differences between the text messages and face-to-face responses. For caregivers who participated in both surveys, we asked them to rate the two methods on a 5-point scale to assess acceptability (1=dislike very much, 2=dislike, 3=ok, 4=like, 5=like very much) [34]. After the interview, the supervisor checked the completeness of all the questionnaires. We gave a towel, worth ¥2.0 (US $0.32), to the participants for their participation.

### Questionnaire

As shown in Textbox 1, the first question participants were asked was about informed consent (only in text messaging survey); there were an additional 7 core questions in both surveys. Participants were not sent any other questions/text messages if they replied "no" to the informed consent question. The 7 core questions were divided into 3 categories: participants’ feedback on the infant feeding calendar, participants’ feeding knowledge, and the main source of their feeding knowledge. Two questions about participants’ feeding knowledge were from the Breastfeeding and Nutrition module of the Maternal, Newborn, and Child Health household survey developed by the WHO; we developed the other 5 questions. All these questions were tested in the pilot study and revised according to the feedback of caregivers.

The text messaging survey consisted of 11 messages: 2 introduction messages which did not need replies, 1 informed consent message which asked whether the participant was willing to participate, 7 messages with the 7 core questions, and 1 thank you message. In the introduction messages, we informed participants who we were, what the aim of the study was, and what benefits (an infant feeding recipe) they could get after completing the text messaging survey.

The face-to-face survey had 27 questions in total: 10 questions about general information of children and participants, the 7 core questions, 7 questions about reasons for disagreement between the same questions, one question about reasons for nonresponse or incomplete replies, and 2 questions about participants’ perceptions of the 2 survey methods.

**Text messaging survey content.**

Text message 1: Hello! This is the Capital Institute of Pediatrics and Zhao County Maternal and Child Health Hospital. We have given you a feeding calendar and now would like to know how you use the feeding calendar.

Text message 2: This survey does not require extra expenses apart from your normal text message costs, which is 1 jiao per message. Please reply to our text messages. Caregivers who reply to all these messages will get a feeding recipe in a couple of days (do not reply to the previous 2 messages).

Text message 3 (question 1): Please reply to questions one by one. After replying to a question, you will receive the next question. There are 8 questions in total. Do you agree to participate in the survey? Please write the number of your choice below, and then send the message.

1. Yes
2. No

Text message 4 (question 2): Have you or other members of the family read the feeding calendar? Please write the number and send.

1. Yes, I have read it carefully
2. Yes, I have read it
3. Yes, I have read part of it
4. No, I have not read it
5. No, I did not get it

Text message 5 (question 3): In your opinion, is it easy for you to understand the feeding knowledge? Please write the number and send.

1. Yes, it is very easy.
2. Yes, it is easy.
3. Yes, it is ok.
4. No, it is difficult.
5. No, it is very difficult.

Text message 6 (question 4): In your opinion, is the feeding knowledge calendar useful for you to feed your child? Please write the number and send.

1. Yes, it is very useful.
2. Yes, it is useful.
3. Yes, it is ok.
4. No, it is useless.
5. No, it is completely useless.

Text message 7 (question 5): In your opinion, until what age should a child be given only breast milk, without any other food or liquids (including water)? Please write your answer in months, and send.

Text message 8 (question 6): In your opinion, at what age should a child be given meat (such as pork, beef, mutton, chicken, and duck)? Please write your answer in months, and send.

Text message 9 (question 7): In your opinion, until what age should a child be breastfed? Please write your answer in months and send.

Text message 10 (question 8): Where did you receive this feeding knowledge? Please choose the most important one below. Please write the number and send.

1. The feeding calendar
2. Township hospital doctors
3. Village doctors
4. By yourself
5. Relatives or friends
6. Other

Text message 11: You have completed all questions, thank you for your cooperation!

### Outcomes

The primary outcomes were the response rate, data agreement, and costs. The secondary outcomes were acceptability, reasons for nonresponse to text messages, reasons for disagreement between the 2 survey methods, and the appropriate time to send text messages.

### Response Rate

In both surveys, we defined the response rate in 2 ways: (1) the proportion of participants who answered at least 1 core question, and (2) the proportion of participants who completed all the core questions.

### Data Agreement

Data agreement could only be assessed when the same participant participated in both the face-to-face and text messaging surveys. We reported intraclass correlation coefficient (ICC) and kappa values, and the percentage of agreement of responses that were the same for both methods [35].

### Costs

We assessed the total costs, costs per questionnaire, the costs per completed questionnaire, and the incremental cost effectiveness ratio (ICER), which was calculated for the proportion of participants who completed all the core questions. The costs included expenses for the two methods from 9 sources: transportation, food, hotel, printing, text message, renting a mobile phone, data entry, payments for interviewers, supervisors, and local coordinators, and gifts for interviewees.

### Acceptability of Text Messaging and Face-to-Face Surveys

We defined acceptability as the rating that participants who participated in both surveys gave. We report the median points of caregivers’ acceptability scores (on a 5-point scale; 1=dislike very much, 2=dislike, 3=ok, 4=like, 5=like very much) for both surveys.

### Reasons for Not Responding to Text Messages

Reasons for not responding were reasons participants gave for not replying to text messages.

### Reasons for Disagreement Between the Survey Methods

Reasons for disagreement were reasons caregivers thought may be the reason they gave different answers.

### Appropriate Times to Send Text Messages

A better time to send text messages is the time interval when most text messages are replied to. We defined the best time as the time when at least 75% of text messages were replied to within 1 hour of being received.

### Data Analysis

We entered data for both surveys into EpiData 3.1 (The EpiData Association, Odense, Denmark). We checked the 2 files and resolved discrepancies by checking the original data in the smartphone or face-to-face questionnaires.

We used the McNemar test to detect differences between the survey methods in the overall response rate. We assessed the data agreement by kappa value (simple kappa for categorical variable, Fleiss-Cohen for ordinal data), ICC for quantitative variables, and percentages of the same answers in both methods [36]. We used the pairwise Wilcoxon rank test and kappa value to compare the caregivers’ acceptability for both survey methods. Two team members read and discussed reasons for both nonresponse and inconsistent answers and then classified the answers into different categories. We calculated percentages to describe provided reasons for not responding to the text messaging survey, disagreement in responses between the 2 survey methods, and the appropriate time for sending messages. We used SAS 9.1 (SAS Institute, Cary, NC, USA) for the analysis and we considered a *P* value less than .05 as statistically significant.

### Ethical Approval

We obtained ethical approval from the Ethical Committee of the Capital Institute of Pediatrics in Beijing. For face-to-face survey, all surveyed participants read the informed consent form and gave their written consent. For the text messaging survey, participants gave their consent through a text message.

## Results

### Participants

**Figure 1:**
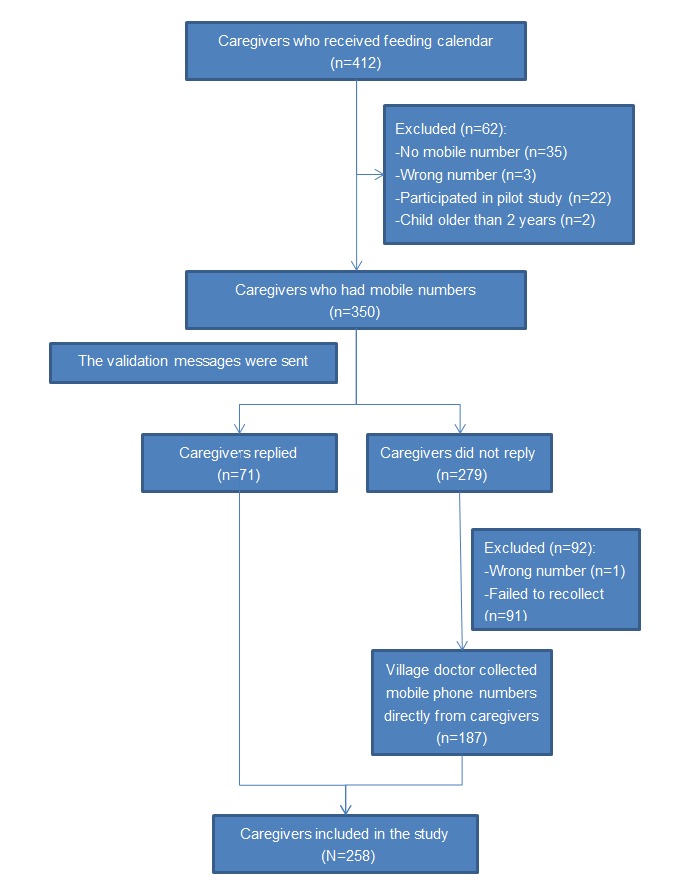
Flowchart of participant enrollment.

Figure 1 shows the flowchart of the enrollment of participants. We included 258 (62.6%) participants in our study out of the 412 caregivers who received feeding calendars. Of those 412 caregivers, we had to exclude 62 (15.0%) caregivers because we did not have the mobile phone number for the following reasons: no numbers (35/62, 56%), wrong numbers (3/62, 5%), participated in pilot study (22/62, 35%), or had children aged older than 2 years (2/62, 3%). We sent validation messages to the remaining 350 caregivers and we were able to validate the mobile phone numbers of 71 (20.3%) caregivers who responded. For the 279 (79.7%) caregivers who did not respond to our messages, village doctors were able to recollect 187 mobile numbers. We compared the numbers they found with our own records, but we did not send text messages again to caregivers to validate the numbers. The remaining 92 caregivers whose mobile numbers were not recollected by village doctors were excluded. In all, a total of 258 caregivers were included in this study.

Table 1 lists the total number of participants in the text messaging survey and face-to-face survey and the characteristics of participants and their children. Among the 258 included participants, 99 (38.4%) participated in text messaging survey, 177 (68.6%) participated in the face-to-face survey, and 43 participants (16.7%) participated in both surveys. The age and sex ratios of the youngest child in families were similar for the 2 surveys. Participants who participated in our surveys included primarily mothers, but also fathers, grandparents, and other family members. There were a higher proportion of grandparents in the face-to-face survey (29.4%) than in the text messaging survey (1.0%).

**Table 1:** Characteristics of survey participants and their children (N=258).

Characteristics		Text messaging survey (n=98)	Face-to-face survey (n=177)
**Children**			
	Age in days, mean (SD)	359.6 (154.5)	359.8 (155.7)
	Sex (male/female)	62/37	116/61
**Participants, n (%)** ^a^			
	Mother	86 (87.8)	115 (65.0)
	Father	10 (10.2)	7 (3.9)
	Grandparent	1 (1.0)	52 (29.4)
	Other	1 (1.0)	3 (1.7)

^a^Data missing for 1 participant in the text message survey group because the interviewer forgot to ask this question.

### Response Rate

**Figure 2:**
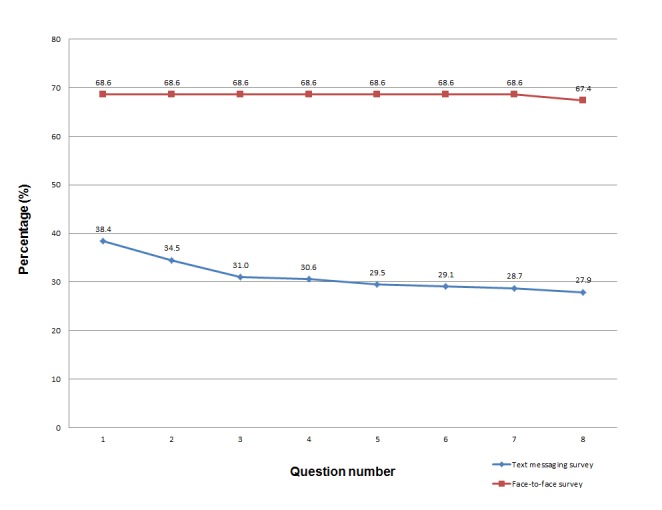
Response rates for each question in the text messaging and face-to-face surveys.

For the text messaging survey, 99 (38.4%) of 258 participants responded to our first question and 72 (27.9%) completed all 8 questions (72.7% of participants who responded to the first question). For the face-to-face survey, 177 (68.6%) participants participated and 174 (67.4%) completed all questions. There was a significant difference in the response rate for the first question between the 2 surveys (McNemar test χ^2^_1_= 46.8, *P*<.001) and also for the response rate for all questions (McNemar test χ^2^_1_= 68.6, *P*<.001). Figure 2 shows the response rates for both surveys.

### Data Agreement

There were a total of 255 questions answered by the same participants in both surveys. Table 2 shows that 159 (62.4%) questions had the same answers for both surveys. The lowest proportion of agreement was for the fourth question, with 19 (56%) of 34 responses having the same answers; the highest proportion of agreement was for the last question, with 28 (85%) of 33 responses having the same for both methods. The Fleiss-Cohen kappa values and ICCs showed a moderate to good agreement for most questions. The Fleiss-Cohen kappa values were .68 (95% CI 0.43-0.92), .50 (95% CI 0.21-0.79), and .23 (95% CI –0.12 to 0.58) for ordinal data (questions 2-4), respectively. The ICC for quantitative data (questions 5-7) were .53 (95% CI 0.29-0.76), .72 (95% CI 0.51-0.86), and .69 (95% CI 0.50-0.83), respectively. Simple kappa for categorical data (question 8) was .76 (95% CI 0.56-0.96).

**Table 2:** Questions with the same answers in both surveys by the same participants.

Question	Questions by same person, n	Questions with same answers, n (%)	Survey method, median (interquantile range)		Kappa/ICC (95% CI)
			Face-to-face	Text messaging	
2	43	25 (58)	2.00 (1.00, 2.00)	2.00 (1.00, 2.00)	.68 (0.43, 0.92)^a^
3	35	23 (66)	2.00 (1.00, 3.00)	3.00 (1.00, 3.00)	.50 (0.21, 0.79)^a^
4	34	19 (56)	1.00 (1.00, 2.00)	1.50 (1.00, 2.00)	.23 (–0.12, 0.58)^a^
5	29	18 (62)	6.00 (5.00, 6.00)	6.00 (5.00, 6.00)	.53 (0.29, 0.76)^b^
6	29	19 (66)	7.00 (6.00, 8.00)	6.00 (6.00, 7.00)	.72 (0.51, 0.86)^b^
7	31	22 (71)	18.00 (12.00, 24.00)	18.00 (12.00, 24.00)	.69 (0.50, 0.83)^b^
8	33	28 (85)	—	—	.76 (0.56, 0.96)^c^
Total	255	159 (62)	—	—	—

^a^Fleiss-Cohen kappa

^b^ICC

^c^Simple kappa

### Costs

The costs shown in Table 3 are all directly related to the data collection project. The total costs of the face-to-face survey were much higher than the costs of the text messaging survey: ¥5993.1 (US $953.12) and ¥1954.5 (US $310.84), respectively. Costs per questionnaire for the face-to-face survey were also higher than for the text messaging survey: ¥33.9 (US $5.39) and ¥19.7 (US $3.13) for all questionnaires, respectively, and ¥34.4 (US $5.47) and ¥27.1 (US $4.31) per completed questionnaires, respectively. Table 3 shows the total costs and costs per questionnaire for both surveys. The ICER was calculated to be ¥102.2 (US $16.25), meaning the cost of the face-to-face survey for each percentage increase of completion rate was ¥102.2 (US $16.25) compared to the text messaging survey.

**Table 3:** Cost^a^ comparison of both surveys.

Item	Face-to-face (¥/US $)	Text message (¥/US $)
Transportation	1606.0 (255.41)	0.0 (0.00)
Food for interviewer and supervisor	600.0 (95.42)	300.0 (47.71)
Hotel for interviewer and supervisor	1440.0 (229.01)	0.0 (0.00)
Printing of questionnaires and informed consent form	150.0 (23.86)	0.0 (0.00)
Text message	0.0 (0.00)	166.8 (26.53)
Renting mobile phone	0.0 (0.00)	150.0 (23.86)
Data entry	53.1 (8.44)	29.7 (4.72)
Payment for interviewers, supervisor, and local coordinators^b^	1790.0 (284.67)	1110.0 (176.53)
Gift for participants	354.0 (56.30)	198.0 (31.49)
Total	5993.1 (953.07)	1954.5 (310.84)
Per questionnaire (all questionnaires)	33.9 (5.39)	19.7 (3.13)
Per questionnaire (completed questionnaires)	34.4 (5.47)	27.1 (4.31)

^a^Based on ¥/US $ exchange rate on April 15, 2012.

^b^We asked township health workers and village doctors as local coordinators to collect and validate the mobile phone numbers for text messaging survey and to recruit interviewees for face-to-face survey.

### Acceptability of the Text Messaging and Face-to-Face Surveys

Participants who participated in both surveys gave their acceptability scores for both surveys. As indicated in Table 4, the medians for both the text messaging and face-to-face surveys were the same (median 4) and there was no significant difference between the surveys (Wilcoxon signed rank test=15, *P*=.41, κ =.512, 95% CI 0.301-0.724).

**Table 4:** Participants’ perceptions of the text messaging and face-to-face surveys (N=43).

Perceptions^a^		Text messaging survey, n			Total
		3 (ok)	4 (like)	5 (like very much)	
**Face-to-face survey, n**					
	3 (ok)	3	2	1	6
	4 (like)	3	12	1	16
	5 (like very much)	1	5	15	21
Total		7	19	17	43

^a^For both surveys, no participants chose 1 (dislike very much) or 2 (dislike).

### Reasons for Not Responding to Text Messages

A total of 159 participants did not reply to our text messages. Of these, 104 (65.4%) participated in the face-to-face survey. Of the 104 participants, 51 (49.0%) were not the holder of mobile phones to which we sent the text messages. We asked the remaining 48 participants about their reasons for not replying to our text messages (data missing for 5 caregivers because interviewers forgot to ask this question). Table 5 indicates that 19 of 48 (40%) caregivers reported that they did not receive our text messages, and 16 (33%) of them reported that they were too busy to reply or did not see the text message in time.

**Table 5:** Participant reasons for nonresponse to text messages (n=48).

Reasons	n	%
Did not receive text message	19	40
Too busy to reply or did not see text messages in time	16	33
Did not know how to reply	5	10
Did not want to reply	4	8
Other^a^	2	4
Do not know	2	4

^a^One “did not know that this message needed a reply” the other “forgot to reply.”

### Reasons for Disagreement Between Survey Methods

There were 43 participants who answered 255 questions in total: 159 (62.4%) questions with the same answers and 96 (37.6%) questions with different answers when comparing the face-to-face and text message questions. We asked participants for the reasons for disagreement. We obtained 69 answers; participants did not provide their answers for the remaining 27 questions. Table 6 indicates that for questions with different answers, approximately two-thirds (67%) of participants said they had forgotten their text message answers in face-to-face interview, 1 in 6 participants (17%) changed their answers, and 1 in 10 participants’ (10%) said that they misunderstood the text message question.

**Table 6:** Reasons for disagreement between the survey methods (n=69).

Reasons	n	%
Forgot the answer to this question	46	67
Changed ideas	12	17
Writing wrong numbers because misunderstood question in text messaging survey	7	10
Gave wrong answer because misunderstood question in face-to-face survey	2	3
Do not know	2	3

### The Appropriate Time to Send Text Messages

**Figure 3:**
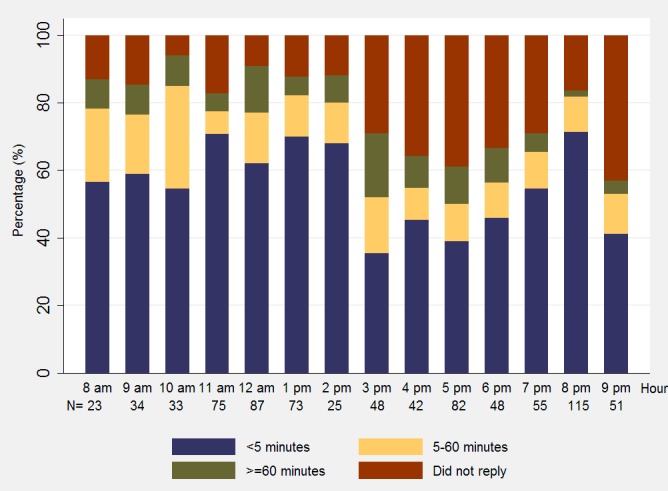
Response times to text messages between 8 AM and 9 PM by hour.

The smartphone automatically recorded the time sent and the reply time of each text message. Instead of sending all questions to participants at the same time, we sent the next question only after we received a reply message to the previous question; therefore, some questions were not sent to all participants because of nonresponse for the previous question. We sent 806 text messages in total and received 628 reply messages from caregivers. Figure 3 shows the time interval between sending and replying to messages across the day (each column represents 1 hour). In this chart, we excluded 10 pm to 11 pm because only 7 messages were sent at 10 pm and 8 messages at 11 pm. Participants responded quicker from 8 am to 3 pm and from 8 pm to 9 pm. During these periods of time, more than 50% of the replied text messages were replied to within 5 minutes and more than 75% were replied to within 60 minutes.

## Discussion

### Principal Results

Our study showed the text messaging method had a lower response rate, moderate to good data agreement, and lower cost compared to the face-to-face method. Participants who participated in both surveys found the methods similarly acceptable. The main explanations the participants provided for not replying were not receiving text messages, being too busy to reply, or not seeing the text message in time. The main explanations provided by the participants for disagreement were that participants forgot their text message answers and they changed their minds. We also found that participants were more likely to reply immediately after they received text messages in 2 time periods: from 8 am to 3 pm and between 8 pm and 9 pm.

### Comparison With Prior Work

A low response rate of a survey sample leads to nonresponse error; therefore, it is a key issue for text messaging data collection. Studies in the literature indicated different response rates, varying from 14% to 100% [12,15-17,37-41]. A study with 2400 randomly selected mobile phone numbers from the Swedish population registry achieved a response rate of 14% [37]. Another study with a small sample size had a response rate of 100% [15]. The response rate in our study was modest, 38.4% for the first question (consent) and 27.9% for all 8 questions in the survey. Among 99 caregivers who replied to the first text message, 74 (74.7%) of them completed all 8 questions. It is promising that interviewees are more likely to complete the text messaging survey when they respond to the first text message question, but the initial response is a key to recruit interviewees. Therefore, there is a need to explore how the response rate can be increased.

Many factors can affect the response rate of text messaging surveys. The initial contact with participants is the first step; however, this often fails because of difficulties in finding functioning mobile phone numbers [37]. An advance letter to introduce the aim and meaning of the survey, benefits to participants, and who the surveyors are could contribute to a higher response rate [42], whereas a foreign phone number might decrease the trustworthiness and lower the response rate [37]. Participants’ awareness of the survey and their trust in the surveyors can increase their willingness to respond [15,37]. We asked village doctors, whom most caregivers trust, to verbally inform interviewees in advance, and we sent an introduction message. Participants’ professions and education levels also affect the response rate. The participants of the study that had a 100% response rate were undergraduate college students [15], whereas the collection of human immunodeficiency virus (HIV) treatment adherence data by text messaging from HIV-infected children’s caregivers who only completed primary school in Uganda indicated very low response rate [16]. In our study, participants were caregivers, including parents and grandparents, in rural areas and most were farmers with a junior high school education. The illiteracy rate for people aged 15 years and older was 4.07% in rural areas in Hebei province in 2010 [43]. In our pilot study, we did not find that illiteracy was a problem and we expected our target study population was mainly literate. However, this may be different in other study settings. Our pilot study showed that most caregivers could reply to messages, but in our study, approximately 10% of those who did not respond reported that they did not know how to reply to the text messaging survey. This has to be taken into consideration for future research. Sending text messages at an appropriate time could also increase the response rate. Some studies on telephone surveys showed that targeting the call time or calling at an anticipated time was an effective strategy to increase response rate [42]. Bexelius et al [44] found that a lower proportion of participants responded to text messages that were sent at 5 pm, which is usually a time when many people are in transit. Some participants in our study reported that they were too busy to reply or they did not see our text messages in time, which meant that our text messages were not sent to them at a convenient time. However, we found that participants responded more quickly between 8 am and 3 pm and between 8 pm and 9 pm, which could potentially be an appropriate time for sending text messages to our study population. Reminders can increase the response rate. In Kew’s study, they sent 3 text message reminders to participants and if that did not work, they reminded participants by calling them [15]. However, in a trial in which a daily reminder message was sent for taking a vitamin C pill for 1 month, only 45% of participants would continue to use it for a longer period of time [45]. Therefore, in our study we sent only 1 reminder message and gave participants 3 days to respond. Further research is needed to test the usefulness and tolerance for reminders in a text messaging survey.

We found that the most important reasons participants’ gave for not responding to text messages were that they did not receive the survey text messages or that they were too busy to reply followed by they did not know how to reply or they did not want to reply. Example interventions to solve these problems are to make more effort to have personal contact with participants, let village doctors inform caregivers more actively, send information messages on the research and how to reply, update mobile phone numbers regularly, and send text message at the appropriate times. Further studies need to be conducted to explore in-depth the reasons for not responding and the effectiveness of these approaches on increasing response rates of text messaging surveys.

Data validity is the prerequisite for text messaging to be accepted as a data collection method, and it has been addressed by a small number of studies. Whitford et al [17] found that a text messaging survey had excellent agreement compared to a telephone interview for collecting information on infant feeding. A study comparing telephone interviews and text message data collection for disease symptom reporting also acquired a high degree of agreement [12]. Our study compared a text message survey with a face-to-face survey and found moderate agreement. However, by exploring the reasons for disagreement between the two methods, we found that nearly 20% of disagreement was because of participants’ changing their minds and only 10% was reported to be directly caused by the text messaging method (primarily because of writing the wrong numbers or misunderstanding questions sent by text messages). Further research needs to be done on a larger sample of participants so that kappa values can be calculated.

The cost of the study would change with different study designs. In our study, the costs could be less if a number of factors changed. Firstly, there were fees for data entry because there was no software that could transfer data from the mobile phone text messages to the computer at that time. Therefore, these costs could be deducted for data entry. Secondly, an automated text messaging system can avoid fees by renting the mobile phone and deducting the messages’ fees. Thirdly, the nonresponse rate and text message reminders increased the average fees for every questionnaire. Therefore, the higher the response rate, the lower the costs of the survey. Fourthly, the fees for text messages and renting mobile phones will decrease with the rapid development of technology. However, we expect that fees for transportation will increase, and that the fees for food and hotel for interviewers and supervisors, printing, data entry, and gifts for caregivers will not change dramatically in China within a relatively short period. Overall, text messaging data collection methods can potentially save money on program monitoring.

### Strength and Limitations

The strength of our study is that we explored the reasons for nonresponse and disagreement between the 2 survey methods. This gave us more insight into why some participants did not respond and why responses were different, and this information could be used for further studies to increase the response rate or validate text messaging surveys. In our study population, the highest responses were achieved from 8 am to 3 pm and between 8 pm and 9 pm, which can guide future text messaging data collection. However, our study had some limitations. First, we used a mobile phone to manually send text messages because we could not find an appropriate text message platform at the time of the study. Second, our sample size for data agreement analysis was small because of the low response rate of the text messaging survey. The reasons for nonresponse and responding differently in the 2 survey methods were asked face-to-face and provided by caregivers, but we have no way to verify their answers. Interviews might be needed to explore the real reason for nonresponse and inconsistent answers.

### Conclusions

To our knowledge, this is the first study that explored the feasibility of using text messaging as a data collection method for program monitoring in rural China. Although the text messaging survey was acceptable to interviewees who participated in both surveys and costs were lower than for the traditional face-to-face method, it had a lower response rate than the face-to-face method. Future research needs to evaluate the effectiveness of strategies that can increase the response rate, especially in collecting longitudinal data by text messaging.

## Abbreviations

HIV: human immunodeficiency virus
ICC: intraclass correlation coefficient
ICER: incremental cost effectiveness ratio
WHO: World Health Organization
